# Screening of peptide probe binding to particulate matter with a high metal content†

**DOI:** 10.1039/c7ra13290e

**Published:** 2018-02-06

**Authors:** Masayoshi Tanaka, Aw Wei Liang Alvin, Mina Okochi

**Affiliations:** Department of Chemical Science and Engineering, Tokyo Institute of Technology 2-12-1, O-okayama, Meguro-ku Tokyo 152-8552 Japan okochi.m.aa@m.titech.ac.jp +81-3-5734-2116 +81-3-5734-2116

## Abstract

Particulate matter (PM) is becoming an increasing health concern and there is a need to develop detection methods to keep its harmful effects in check. Generation of reactive oxygen species (ROS) by PM is often associated with metal compounds, hence our aim is to screen for a peptide probe towards improved collection and the detection of PM having a high metal content. Peptides are putative recognition molecules due to their versatility and ease of modification to enhance their binding selectivities. PM binding peptides were screened using the peptide array and different binding behaviors in terms of different spot colors (yellow, mixed and gray), indicating the different composition of bound PMs, were observed. The strongest binding peptides were identified as follows: NHVNTNYYPTLH (gray), NGYYPHSHSYHQ (mixed) and HHLHWPHHHSYT (yellow), with relative binding ratios of 125%, 144% and 136%, in comparison with WQDFGAVRSTRS, a peptide screened from a phage display in our previous study. Inductively coupled plasma mass spectrometry (ICPMS) analyses revealed that Co, Ni and Zn content in the PM bound to the HHLHWPHHHSYT peptide spot were respectively 12.5, 15.8 and 7.8 times that of the PM bound to no peptide spot, suggesting this peptide probe is applicable to collect PM with a high metal content.

## Abstract 2

Using peptide array, peptides binding to particulate matter with high metal content were screened and characterized by focusing on the different spot colors (yellow, mixed and gray).

## Graphical abstract

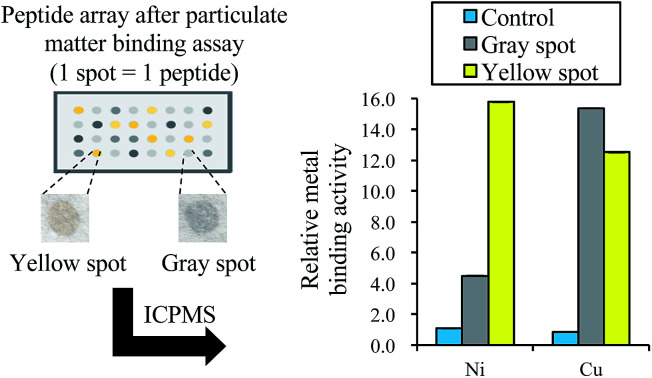

## 1. Introduction

Particulate matter (PM) is defined as fine solid or liquid matter suspended in a gaseous medium.^1^ PM is a complex mixture containing elemental and organic carbon (EC and OC), inorganics such as sulfates and nitrates, as well as metal compounds.^2–4^ PM is an important metric for both indoor and outdoor air quality^5,6^ and a major source of health concern in urban areas.^7^ Short term exposures to fine PM increase the risks of myocardial infarction, stroke and heart failure exacerbation^8^ while long term exposures have been associated with increased mortality rates.^9,10^ Fine PM mortality costs per tonne of inorganic air pollutants in the United States were estimated to be USD$88 000–130 000 and the costs for 2005 emissions totalled USD$1.0 trillion dollars.^11^ The social and economic costs of PM emissions have become the motivation for various innovation efforts to develop PM detection devices and *in vitro* experiments to prove the mechanisms underlying PM toxicity.

Reactive oxygen species (ROS) is a collective term to refer to oxygen and hydroxyl radicals, as well as reactive derivatives of oxygen such as hydrogen peroxide and singlet O_2_.^12^ In healthy organisms, the rate of generation of ROS is more or less balanced by anti-oxidant activities and radical scavenger defenses.^12^ However, when the prooxidant/antioxidant equilibrium shifts in favour of the former, a phenomenon known as oxidative stress ensues.^13,14^ Oxidative stress causes oxidative damage to proteins, membranes and genes, and leads to the development of cardiovascular diseases, neuronal degeneration and cancer.^15^ Many studies have shown the ability of PM to generate ROS and induce oxidative stress in the body when inhaled,^16–19^ hence oxidative stress is hypothesized to be one of the key mechanisms underlying PM toxicity.^20^

The ability of PM to induce ROS responses is often attributed to specific PM components. Among them, metal compounds, soluble and insoluble, have been found to have positive correlations with ROS activity in various *in vitro* studies. Identified water soluble metal compounds include Cu,^21–23^ Fe,^24^ Mn,^23,24^ Co,^24^ Ni,^22,24^ As,^22,23^ Zn^22,23^ and Cr^22^ while identified water insoluble metal compounds include Pb,^21^ Ni,^21^ Fe,^21^ Cr^21^ and Zn.^25^ Although both soluble and insoluble metal compounds are associated with ROS generation, ROS responses due to insoluble metal compounds tend to be prolonged because insoluble compounds are removed more slowly than soluble compounds.^26^

Given that the chemical composition of PM plays a huge role in determining its ROS activity/toxicity, component-based detection methods capable of distinguishing PM with high ROS activities from PM with low ROS activities are desirable. In that regard, peptides serve as good recognition molecules because they display a large variety in terms of physical and chemical properties. As a result of this diversity, they are able to bind to various targets or respond to external stimuli.^27–31^ In addition, peptides can be engineered to modify their binding behaviors and improve their binding specificity towards a given target. The potential of the application of peptides to detect and distinguish PM having different metal compositions was illustrated in our previous study, where we successfully identified four PM binding peptides using phage display and evaluated the differences in the composition of the PM bound to these peptides using inductively coupled plasma mass spectrometry (ICPMS).^32^ In particular, differences in binding properties towards Cu and Zn compounds in PM were observed due to the different proportions of histidine (H) residue in the peptides.

The aim of this study is to expand on our previous work and screen for a peptide probe towards collection and detection of PM with high metal content. Special attention was given to the peptides' ability to recognize and bind to insoluble metal compounds in PM due to the ability of insoluble metal compounds to induce chronic ROS responses. Herein, the peptide probe was screened using the peptide array because of its high throughput and ease of library design.^33,34^ Surprisingly, different spot colors were observed after the PM binding assay, which is an indication of the bound PM having different chemical compositions. Therefore, special focus was given to classify the peptides according to their spot color and characterize them in terms of their isoelectric point (pI) and hydrophobic value (GRAVY). The use of peptides as recognition molecules may be useful in the development of a simple and cost-effective device that is capable of achieving real-time detection due to their synthesizability and stability.

## 2. Experimental

### 2.1. Peptide array synthesis

PM binding peptide candidates were screened using a peptide array containing a pre-designed library of peptide sequences. A cellulose membrane (grade 542; Whatman, Maidstone, UK) was activated using β-alanine as the N-terminal basal spacer. Activated Fmoc amino acids (0.5 M) were spotted on the membrane, using a peptide auto-spotter (MultiPep RSi; Intavis AG, Köln, Germany) according to the manufacturer's instructions and in all cases double couplings were applied with some modifications.^32,34–36^ After addition of the first residue, Acp(6) used as additional spacers between the peptides and the cellulose membrane, the remaining amino groups were blocked with 4% acetic anhydride. For each elongation step, the membrane was deprotected with 20% piperidine in *N*,*N*′-dimethylformamide and subsequently washed thoroughly with *N*,*N*′-dimethylformamide followed by methanol. After the final deprotection, side-chain-protecting groups were removed with a solution of water, triisopropylsilane and trifluoroacetic acid in a volume ratio of 2 : 3 : 95 for 3 h. Finally, the membrane was washed thoroughly with diethyl ether, methanol and applied for binding assay. Peptide spot size in peptide array was arranged to a diameter of 4 mm for spot color characterization and 6 mm for ICPMS analysis.

### 2.2. Screening of PM binding peptides using peptide array technology

Prior to conducting the binding assay, the membrane was equilibrated in a mixture of tris-buffered saline and 0.1% tween 20 (TBST) buffer (pH 7.5) containing 15 mM NaCl two times for 5 min each. A PM suspension (0.05% w/w) was prepared by mixing PM (NIES CRM no. 28 Urban Aerosols) in TBST buffer containing 15 mM NaCl. Other particulates used in this study were NiO (Sigma Aldrich, Japan, <50 nm), and ZnO (Sigma Aldrich, Japan, <50 nm). This suspension was added to the membrane and the set-up was incubated at room temperature for 16 h with gentle shaking. At the end of the binding assay, the membrane was washed in TBST containing 15 mM NaCl for 30 min three times to remove nonspecific binding. The membrane was scanned and the spot intensities were measured using ImageQuant TL 7.0 (GE Healthcare, Uppsala, Sweden). The screening process including peptide library design was based on repetitive of peptide array synthesis and binding assay for screening of PM binding peptides with higher affinity. In order to effectively screen target material binding peptides, peptide library was designed with reference to a list of high binding peptides in previous round of binding assay. In this study, in the first round of screening, a peptide library containing 85 peptide sequences was generated using program R based on the amino acid distribution of four PM binding peptides to preferentially include amino acids found in PM binding peptides, FHPRLQQDHWLH, WQDFGAVRSTRS, AGYPLSENFYYP and GLHTSATNLYLH, screened from phage display in our previous work.^32^ As the result of binding assay, some distinct binding behaviors, in the form of yellow, gray and the mixed spot peptides, were observed. Then top 10 yellow spot peptides and top 10 gray spot peptides were selected to preserve the variation in binding behavior in further rounds of screening so that peptide properties responsible for each binding behavior could be identified. If some amino acids were null in the selected peptides, the library was modified to include them at a proportion of 0.5% each to maintain sequence diversity. By repeating this screening process, stronger PM binding peptides were screened.

### 2.3. ICPMS analysis of bound PM onto peptide spot

The peptide spots were punched out, dissolved in aqua regia and quantified the metal components using 7700 ICPMS system (Agilent Technologies, Tokyo, Japan). The exact sample preparation procedures for ICPMS analyses were the same as those reported in our previous study,^32^ except that 8 peptide spots were used per peptide instead of 10. Although ICPMS is able to quantify the amount of each metal element present in the sample, it is unable to identify the type of metal compound. To support the ICPMS results for PM, binding assays with pure compounds, NiO (Sigma Aldrich, 99.8% trace metals basis) and ZnO (∼80% Zn basis), were performed followed by ICPMS quantification. Sample preparation procedures including the binding assay condition were the same as in the case of PM except that the peptide spots with bound pure compounds were dissolved in concentrated HNO_3_ instead of aqua regia.

### 2.4. Image analysis of peptide spot colors after PM binding assay

To characterize the yellow and gray spot peptides, a library of 600 peptides was randomly generated using program R based on the amino acid distribution of the phage display peptides. This library was synthesized on duplicate peptide arrays and PM binding assays were performed. The arrays were scanned and RGB profile plots were obtained for every peptide spot using ImageJ. Average R, G and B values were calculated for each peptide spot. The peptide spots were classified using a self-defined parameter (Evaluation Value: EV), which is calculated as follows:
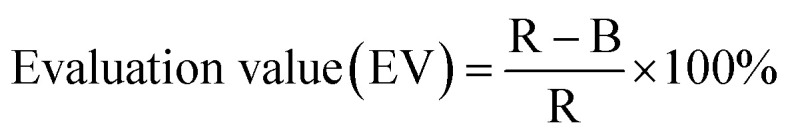


In the RGB color chart, an object appears gray if R ≈ G ≈ B and yellow if R ≈ G > B. The greater the difference between R and B, *i.e.* the greater the EV, the more yellow the peptide spot appears to be.

## 3. Result and discussion

### 3.1. Screening of PM binding peptides using peptide array technology

**Fig. 1:**
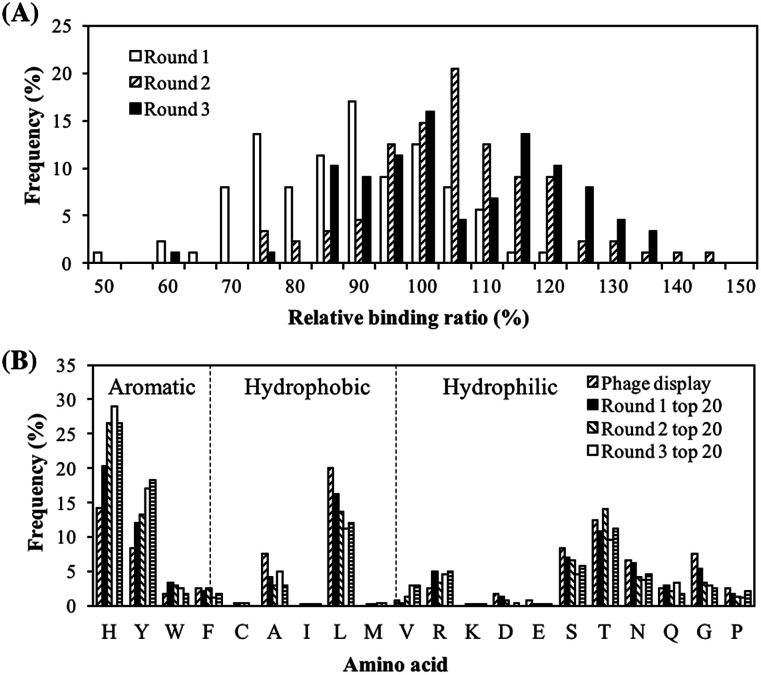
Screening of PM high binding peptides using peptide array and their characterization. (A) Change in relative binding ratios from rounds 1 to 3. The relative binding ratio of a peptide was defined as the ratio of the peptide's spot intensity to the spot intensity of WQDFGAVRSTRS. Relative binding ratio = (peptide spot intensity − background intensity)/(WQDFGAVRSTRS spot intensity − background intensity) × 100%. Background was evaluated at no-peptide spot area. WQDFGAVRSTRS was chosen as the representative of the lead sequences because it had the highest spot intensity among the phage display peptides. (B) Amino acid distributions for top 20 peptides of each round. Amino acid distributions of the original phage display peptides, which were used to design the starting peptide library, were also included in each panel.

The purpose of this screening is to obtain a batch of peptides with good PM binding properties so that they can be characterized to identify the peptide properties important for PM binding. Screening was performed using a peptide array and the peptide libraries were updated every round to reflect the amino acid distribution in the top binding peptides. In the first round of screening, serendipitously, different spot colors, which were an indication of different chemical composition of bound PM, were observed. Top binding peptides of each spot color were then chosen to design the library for the next round of screening so that peptides with good binding properties to various components of PM may be obtained. The PM binding properties of the peptide library in each round of screening, quantified in terms of the peptides' relative binding ratios, are shown in Fig. 1A. Going from rounds 1 to 3, there was a rightward shift in the peak of the histogram, implying that the updated peptide libraries had a greater proportion of strong PM binding peptides. The peak shifted from a relative binding ratio of 90% in round 1 to a relative binding ratio of 105% in round 2, while a negligible shift was observed from round 2 to round 3. The peptides with the strongest PM binding properties had relative binding ratios in the range to 140–150% while the weakest peptides had relative binding ratios of about 50–60%. The negative control, AAAAA, had a relative binding ratio of approximately 10% (not shown in histogram).

The amino acid distributions of the top 20 peptides are shown in Fig. 1B. In addition, the amino acid distributions of the top 20 peptides from the peptide library in each round of screening and the original phage display sequences from our previous study^32^ are also shown so that changes in the amino acid distributions can be tracked. The proportion of H and Y residues increased from rounds 1 to 3. These residues have both hydrophilic and aromatic properties, hence suggesting the importance of hydrophilicity and aromaticity in both types of binding behaviors *i.e.* overall PM binding. On the other hand, amino acids with neither aromatic nor hydrophilic properties, such as A and L, saw a decrease in their proportions as the screening proceeded from round 1 to 3. These results were in line with the observations in our previous study.^32^

The top 5% peptides of their relative binding ratios and spot colors are shown in Table 1. The peptides had relative binding ratios ranging from 125% to 144% improvement in PM binding property over the phage display peptides obtained in our previous study.^32^ The peptides with the highest relative binding ratios corresponding to each spot color were NHVNTNYYPTLH (gray), NGYYPHSHSYHQ (mixed) and HHLHWPHHSYT (yellow). Interestingly, screened peptides as high PM binder dominantly showed yellow spot color. This is probably because histidine residues are important both for the PM binding and yellow color spot display. Further discussion is found in later.

**Table 1:** Relative binding ratios of screened peptides

Sequence	Relative binding ratio^a^ (%)	Spot color
NGYYPHSHSYHQ	144 ± 12	Mixed
HHLHWPHHHSYT	136 ± 7	Yellow
NHYYSHTHTYHG	134 ± 19	Yellow
WHHYFRTTHLTT	134 ± 18	Yellow
HHLFHAVYLNHY	133 ± 16	Yellow
GYHHSTYYTHNH	131 ± 17	Yellow
HLVNRLRYPHVH	128 ± 18	Yellow
LTHVLVHFYYHH	128 ± 7	Yellow
YTYTGLHLLASH	127 ± 6	Mixed
LTAHSHHHYHYA	126 ± 18	Yellow
HHTSGHHSLTLT	125 ± 8	Yellow
YRAYHYLSYRDT	125 ± 7	Yellow
NHVNTNYYPTLH	125 ± 7	Gray

a WQDFGAVRSTRS was defined to have a relative binding ratio of 100%.

### 3.2. ICPMS analysis of bound PM onto peptide spot

**Fig. 2:**
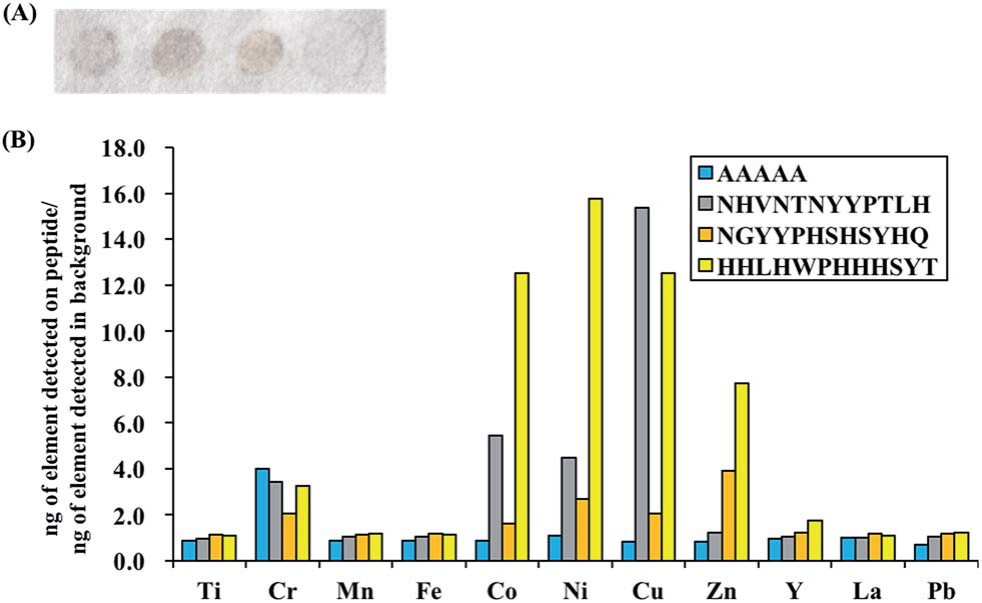
(A) Appearance of peptide spot colors after binding assay with PM: NHVNTNYYPTLH (gray), NGYYPHSHSYHQ (mix), HHLHWPHHHSYT (yellow) AAAAA (control). (B) Ratio of the amount of elements detected in the PM bound to the peptides of interest to those of the background.

To evaluate and compare the composition of the PM bound to the peptides of interest (top gray peptide; NHVNTNYYPTLH, top mixed peptide; NGYYPHSHSYHQ, top yellow peptide; HHLHWPHHHSYT and negative control peptide; AAAAA), ICPMS analyses were carried out for 11 metal elements (Ti, Cr, Mn, Fe, Co, Ni, Cu, Zn, Y, La, Pb) and special binding behaviors were observed for Cu, Co, Ni and Zn. The ratio of the mass of each element detected per unit spot area in the PM bound to each peptide to that of the background spot (no peptide) is shown in Fig. 2. The raw ICPMS data is shown in Fig. S1.† The top gray peptide, NHVNTNYYPTLH, contained the largest amount of Cu compounds. Histidine (H) contains an imidazole group which is able to interact with elemental and partially oxidized Cu surfaces,^37–39^ possibly contributing to the relatively higher Cu compound binding properties in the H-containing peptides. The top mixed color showing peptide, NGYYPHSHSYHQ, contains H residues but it displayed considerably lower Cu compound binding properties compared to the other H-containing peptides. Unlike the other H-containing peptides, NGYYPHSHSYHQ lacked hydrophobic residues, especially L, which was present in all the other H containing peptides. Presence of hydrophobic residues, in addition to H residues, may be an important factor for binding to Cu compounds in PM although this needs to be verified by further experiments. The top yellow peptide, HHLHWPHHHSYT, displayed the strongest binding properties towards Co, Ni and Zn compounds in PM. The Co, Ni and Zn content in the PM bound to HHLHWPHHHSYT were 12.5, 15.8 and 7.8 times of those in the PM bound to the background. From this observation, we believe the yellow color peptides show binding activity to PM with high metal content.

**Fig. 3:**
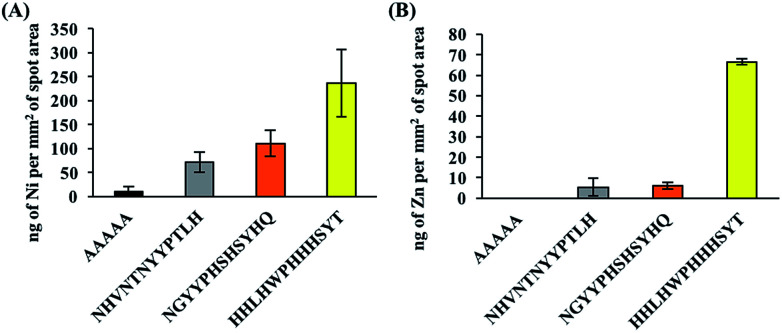
ICPMS measurements, with background subtractions, for (A) Ni and (B) Zn after performing NiO and ZnO binding assays, respectively. Similar to the ICPMS measurements for the PM binding assay, the largest amount of Ni and Zn was detected for the top yellow spot peptide, HHLHWPHHHSYT.

In addition, false positive results, *i.e.* signals of certain elements being detected even though the peptide is not binding to compounds containing those elements, are possible due to the multicomponent nature of PM. To check for the possibility of false positive results, binding assays with NiO and ZnO were also performed and the amount of NiO or ZnO bound to the peptides was quantified using ICPMS. Oxide derivatives were chosen because the top yellow peptide, HHLHWPHHHSYT, contains many positive charges due to histidine residues, a characteristic that is often found in metal oxide binders^40^ and the metal component in PM majorly exists as oxidized metals.^27,28^ ICPMS results for NiO/ZnO binding assays are shown in Fig. 3 and they revealed similar trends to PM binding assays. The top yellow peptide, HHLHWPHHHSYT, had the largest amount of bound NiO and ZnO. It is likely that HHLHWPHHHSYT is detecting and recognizing NiO and ZnO in PM. Many studies have shown positive correlations between the insoluble Ni/Zn compounds in PM and ROS activity,^23,25^ hence there is potential for application of this peptide to detect PM with high ROS activities. It should be noted that the colors of pure NiO and ZnO particles are not yellow. In this study, though we successfully found some peptides with different binding behaviors using spot color-based screening, the direct reason of color differentiation was not specified. Further detail analyses including comprehensive PM component evaluations should be conducted to find out the manner of peptide binding behavior and color differentiation.

### 3.3. Characterization of peptides exhibiting different binding behaviors

**Fig. 4:**
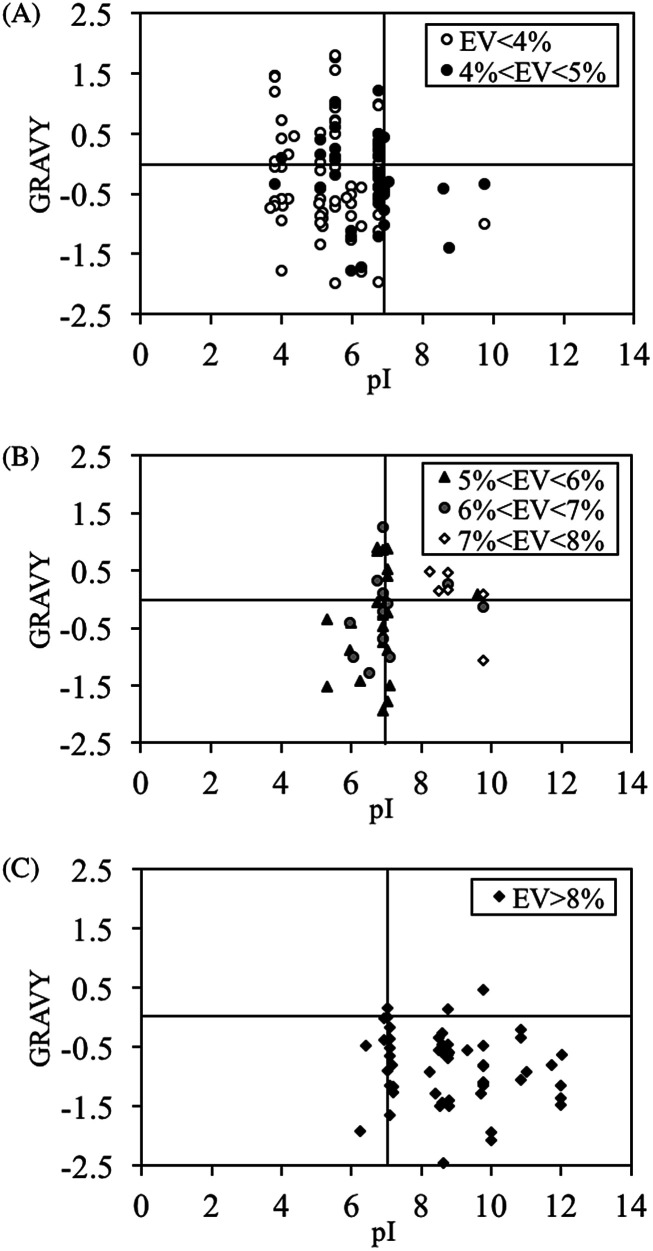
GRAVY and pI characteristics for peptide spots after PM binding with (A) gray, (B) mixed color of yellow and gray and (C) yellow.

To identify the characteristics responsible for each binding behavior that was observed during the screening experiment, a new peptide library containing 600 peptide sequences was generated based on the amino acid distributions of the phage display peptides. After performing a PM binding assay on duplicate membranes containing the aforementioned library, various color spots including gray, yellow and the mixed were found (Table S1†). In order to convert the spot color to numerical form for the clarification of the color difference between yellow and gray, evaluation value (EV) was self-defined (see Materials and methods section). Although this is not a universal parameter used to classify spot color, it seemed to be sufficient to distinguish between yellow (larger EV) and gray colors (smaller EV) in this study (Table S1†). Peptide spots that consistently fell within a given threshold of EV were selected for next characterization. In order to explore the peptide characteristics for spot color differentiation, pI and GRAVY (grand average of hydropathy) values were evaluated for peptide spots that fell within EVs. pI and GRAVY were chosen because they affect the peptide's ability to participate in electrostatic and hydrophobic/hydrophilic interactions, respectively. The pI *versus* GRAVY plots for EV < 4%, 4% < EV < 5%, 5% < EV < 6%, 6% < EV < 7%, 7% < EV < 8% and EV > 8% are shown in Fig. 4. Peptide spots with EV < 5% converged in the acidic region (pI < 7). On the other hand, as the threshold of EV was increased beyond 8%, the data points converged in the hydrophilic (GRAVY < 0) and basic region (pI > 7). These results suggested that the peptide's binding behavior is primarily decided by its pI. The definition of EV < 5% and EV > 8% shall be adopted for gray and yellow spots respectively from this point onwards. Spots with 5% ≤ EV ≤ 8% shall be considered as “mixed”.

**Fig. 5:**
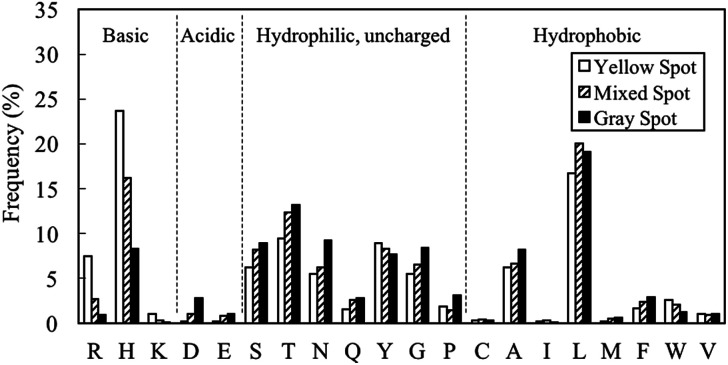
Amino acid distributions for (i) yellow spots (EV > 8%) (ii) mixed spots (5% ≤ EV ≤ 8%) and (iii) gray spots (EV < 5%). Basic residues like R, H and K tend to appear in yellow spot peptides while L and T residues, as well as acidic residues like D and E, tend to appear in gray spot peptides.

The amino acid distributions for gray (EV < 5%), mixed (5% ≤ EV ≤ 8%) and yellow spots (EV > 8%) were investigated (Fig. 5). Basic residues like H, R and K tended to appear in yellow spot peptides. These basic residues are also hydrophilic, hence contributing to the pI and GRAVY distribution observed in Fig. 4C. As shown in Fig. 1B, the H residue was important for PM binding property. However, these characterization results suggested that if the proportion of H residues is increased such that the peptide becomes overall basic, there will be a change in binding behavior of the peptide, which is manifested in the form of a change in spot color from gray to yellow. On the other hand, the proportions of L, T, S, N, Q, G, P, A, F, D and E residues were higher in gray spot peptides than in yellow spot peptides. The residues having the largest proportions, L and T, have pI of less than 7, hence contributing to the acidic nature observed in gray spot peptides. L and T were the dominant residues in hydrophobic and hydrophilic gray spot peptides, respectively. In a review article written by Sarikaya *et al.*^40^ it was observed that metal oxide binders exhibited strong basic characteristics and high positive charges (H and R residues). It can be considered that the yellow spot peptides in this study displayed similar characteristics to the metal oxide binders.

In order to further characterize the peptides exhibiting different binding PM bound colors, substitution assays were performed to confirm the hypothesis found in above experiments; H and R residues tend to be dominant in yellow spot peptides while L and T residues tend to be dominant in gray spot peptides (Fig. S2 and S3†). Herein, since pI was suggested to be the primary factor that determines the peptide's binding behavior, all basic (H, R, K) and acidic (D, E) residues were included in the substitution assays using peptide array. In addition, L and T, which were the dominant hydrophobic and hydrophilic residues, respectively in the gray spot peptides (Fig. 5), were also included in the substitution assays.

The L and T residues in GLHTSATNLYLH were replaced by H, R and K residues and the effects on the EV were evaluated. The EVs of the original sequence and the substituted peptides are shown in the plot in Fig. S2.† The substituted peptides were arranged in order of increasing substitution on the *x*-axis. In all cases, the EV increased with increasing substitution. Similarly, a H/R substitution was performed on the L and T residues in HLVNRLRYPHVH and the effects on the EV were evaluated (Fig. S3†). In all cases, the EV decreased with increasing substitution. These results supported the observations from the characterization of different spot color peptides. There was a loss in PM binding activity (no peptide spots were observed) when the degree of substitution became too high. For T substitutions, loss in PM binding activity occurred from the 4^th^ degree of substitution onwards. In the case of D and E substitutions, loss in PM binding activity occurred from the 3^rd^ degree of substitution onwards. However, in the case of H/R/K substitutions, no loss in binding activity was observed up to the 5^th^ degree of substitution. This suggests that H, R and K residues have stronger PM binding activities than T, D and E residues.

Regarding to the amino acid sequence, within 10 strong yellow spot peptides listed in Table 1, 9 sequences had the H[X*n*]H motif (H: histidine, X: any amino acid, *n*: 1–10 amino acid residues). The observation suggested that the peptides show higher affinity for metal components in PM when multiple H residues are not in a continuous configuration (*e.g.*, not as a multi-residue repeat). The gap between the amino acids may provide a space for the residues to interact effectively with metal surface. Therefore, in addition to the charge and hydrophobicity based characteristics as discussed above, binding manner derived from H[X*n*]H motif may function for the strong binding of peptide probes to PM containing high-metal components. Further analysis would be attained to clarify these hypotheses. In addition, in this study, the peptide probes were designed using natural amino acids. By the modification with other chemicals (*e.g.* terpyridine) and/or unnatural amino acids related to metal binding,^41,42^ peptide probes with improved binding affinity and selectivity may be found.

In this study, the peptides were allowed to interact with PM in suspension, *i.e.* insoluble PM, hence the actual application of the identified peptides, in particular, HHLHWPHHHSYT, for PM detection would ideally require the PM sample to be in suspension form with all soluble components dissolved. In a previous study conducted by Wang *et al.*^43^ a high flow rate particle-into-liquid collector, which is a device that directly collects ambient PM into a concentrated slurry, was successfully developed and coupled with a cupric ion selective electrode to quantify the amount of soluble copper present in the PM sample in real time. In a similar fashion, the particle-into-liquid sampler system can be coupled with peptides mounted on a transducer for real time detection of PM with high ROS activities.

There is potential application of these peptides in filters to capture ambient PM directly even if the soluble components of PM are not dissolved because the peptides can possibly interact with undissolved soluble components of PM, although this needs to be verified with further experiments. Filters modified with pollen specific antibodies to capture pollen directly from the air have been developed,^44^ suggesting the possibility of similar applications in the case of PM using the binding peptides.

## 4. Conclusions

In this study, using peptide array, peptides binding to PM with high metal content were screened and characterized by focusing on the different spot colors (yellow, mixed and gray). In particular, Co, Ni and Zn content in the PM bound to HHLHWPHHHSYT peptide spot were respectively 12.5, 15.8 and 7.8 times of the PM bound to no peptide spot. Since ROS derived from metallic components in PM is known to effect on human health, the functional peptide probe will be useful in the development of environmental technologies comprising the sensing and filtration of toxic PM.

## Conflicts of interest

There are no conflicts to declare.

## Supplementary Material

RA-008-C7RA13290E-s001
