# Supplementary material for: Screening of peptide probe binding to particulate matter with a high metal content
Source: RSC Adv. 2018 Feb 6;8(11):5953–9. doi: 10.1039/c7ra13290e (PMC9078189; doi:10.1039/c7ra13290e)
Supplement: RA-008-C7RA13290E-s001 [file RA-008-C7RA13290E-s001.pdf]

Electronic Supplementary Material (ESI) for RSC Advances.
This journal is © The Royal Society of Chemistry 2018

Supplementary information for:

"Screening of Peptide Probes Binding to Particulate Matter with High Metal Content"

M. Tanaka, A. W. Liang Alvin and M. Okochi *

Department of Chemical Science and Engineering, Tokyo Institute of Technology

2-12-1, O-okayama, Meguro-ku, Tokyo 152-8552, Japan

* Corresponding author: Prof. M. Okochi (okochi.m.aa@m.titech.ac.jp)

Table S1. R, G, B and D values of selected peptide spots.

	Peptide sequence	R	G	B	EV (%)
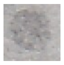	STHLGLLHDLYT	157.48	153.98	154.12	2.13
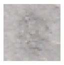	HPDAQNLAGTTS	172.26	168.77	168.54	2.16
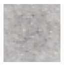	LLNDFTQGSHYG	174.86	171.18	170.51	2.48
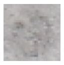	LSGLLTHGLTSL	166.33	162.20	162.12	2.53
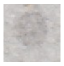	DSTGNPLLRGAT	181.63	177.18	176.19	3.00
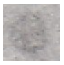	YASLMYPALNGA	163.31	158.81	158.25	3.10
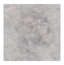	GLNANVEHFSRN	175.72	171.50	169.55	3.51
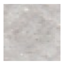	LSADFDHQWGYA	193.13	188.64	186.25	3.56
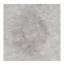	NHLQLGDSNGHT	177.72	172.57	170.61	4.00
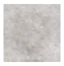	HLPELTHDGRHQ	193.82	189.04	185.94	4.06
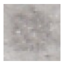	LLSGNSGPHLNW	167.66	161.95	160.12	4.50
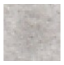	TGEFVLLLHSHQ	183.46	178.11	175.21	4.50
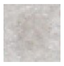	NNPHSLFTAVLH	193.36	187.63	183.70	5.00
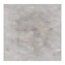	RLLTPRDAGLTL	176.25	170.70	167.38	5.04
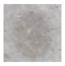	LLHHLLHLLYST	164.16	158.21	155.05	5.55
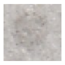	GALNPLLTALAT	179.34	173.21	169.37	5.56
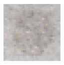	HYGTGATLHWLD	175.85	169.29	165.27	6.02

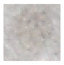	HYLWSGALTGFT	184.05	177.60	172.94	6.04
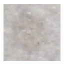	SGSRNNLIHLLS	184.90	178.38	172.87	6.51
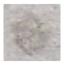	NGSHLGHLTLST	173.80	166.62	162.41	6.55
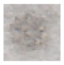	GGFTHASLSHHF	165.62	158.51	154.01	7.01
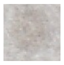	HLLTAGTHGNHA	187.07	179.51	173.83	7.08
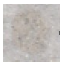	AGAFTSLLHHKL	182.61	175.39	168.90	7.50
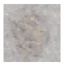	FTRATTSTGKHN	172.19	164.18	159.22	7.53
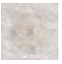	LVLGLLWRGAPT	171.39	163.44	157.66	8.01
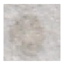	TSLGHHHNYNAH	189.88	182.01	174.64	8.02
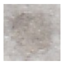	TYLHVNHLNLRA	170.84	162.33	156.37	8.47
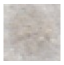	LLFMFNCTGRGT	187.84	179.52	171.85	8.51
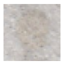	LYFLTTTRLLYY	188.69	180.17	171.82	8.94
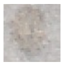	HTHWSTHGHNNH	182.48	173.72	166.01	9.02
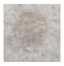	HYVYGLNLQHYG	177.13	167.58	160.43	9.43
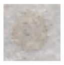	WLPSRLGGFSLH	183.65	174.34	166.16	9.52
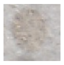	TRHYYPHASLAL	181.68	171.80	163.48	10.01
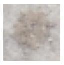	RTSHTPSSNHST	172.22	162.34	154.88	10.07

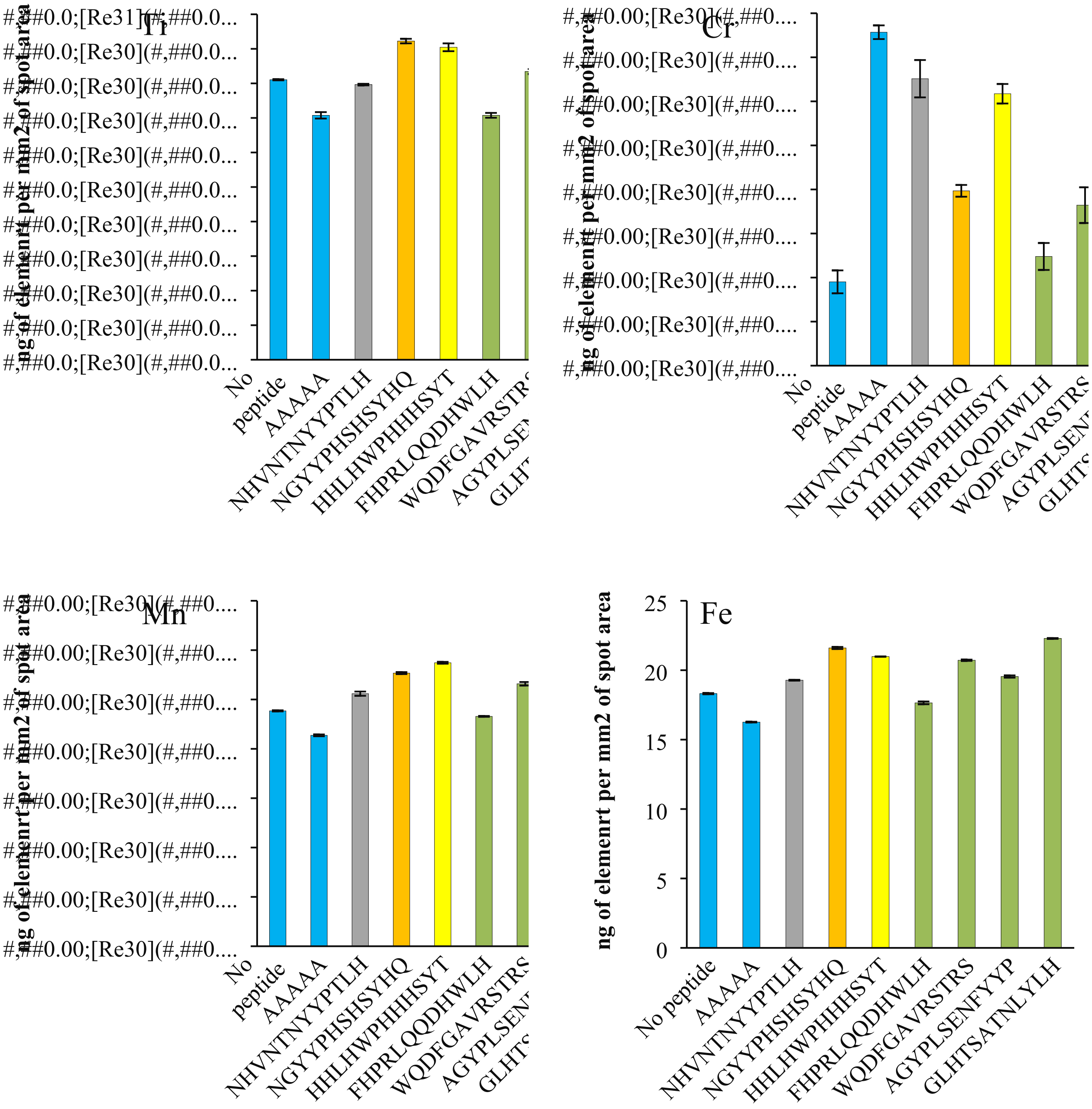

Figure S1. ICPMS analyses for Ti, Cr, Mn, Fe, Co, Ni, Cu, Zn, Y, La and Pb.

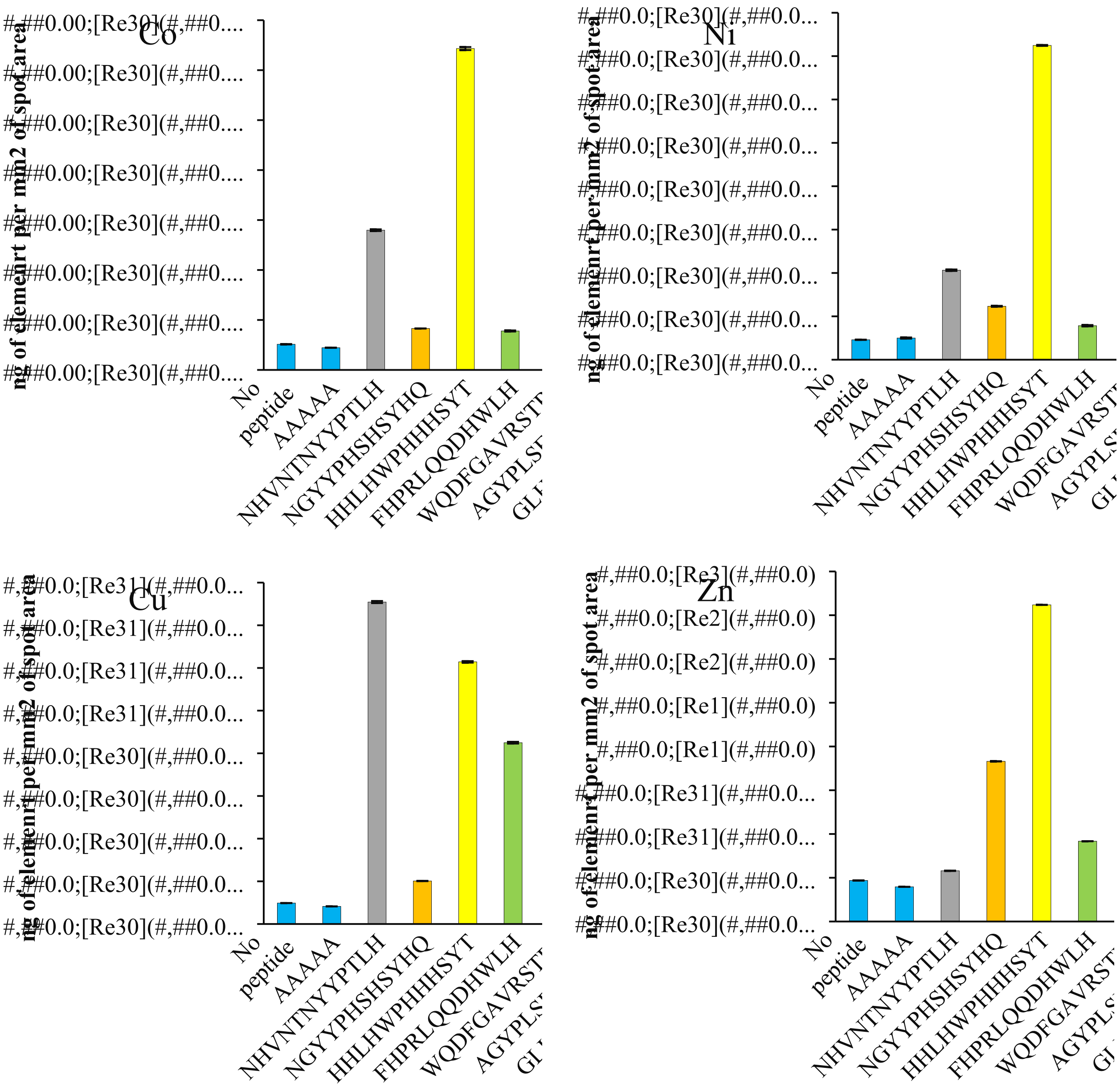
Co
Ni
Cu
Zn
ng of elemenrt per mm2 of spot area
No peptide
AAAAA
NHVNTNYYPTLH
NGYYPHSHSYHQ
HHLHWPHHHSYT
FHPRLQQDHWLH

Figure S1. ICPMS analyses for Ti, Cr, Mn, Fe, Co, Ni, Cu, Zn, Y, La and Pb. (Continued)

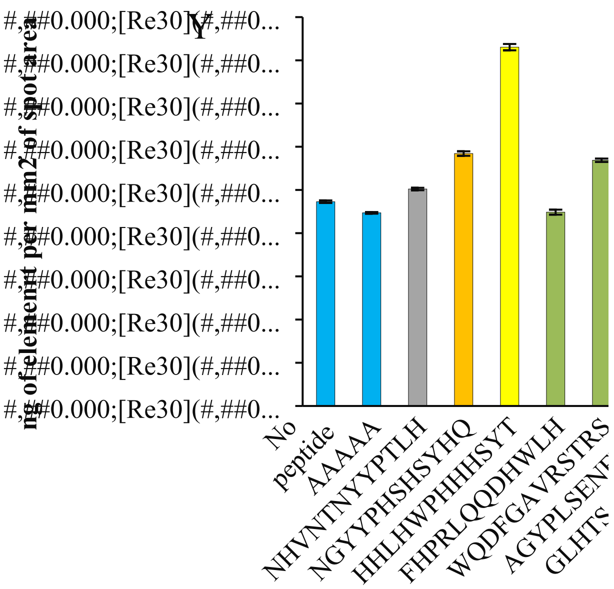

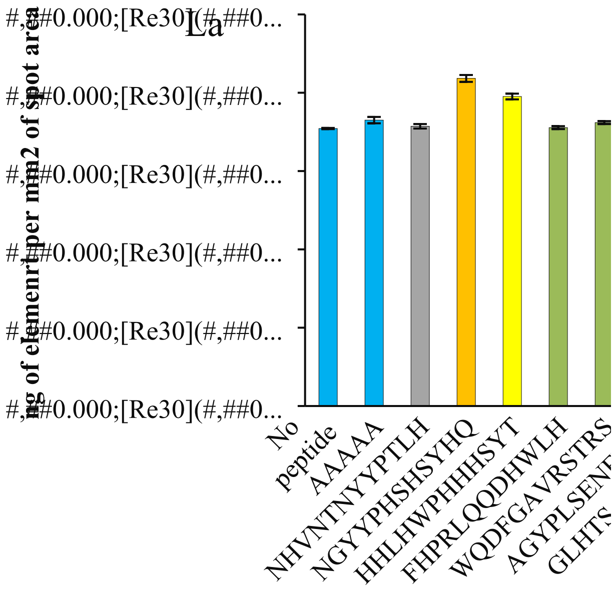

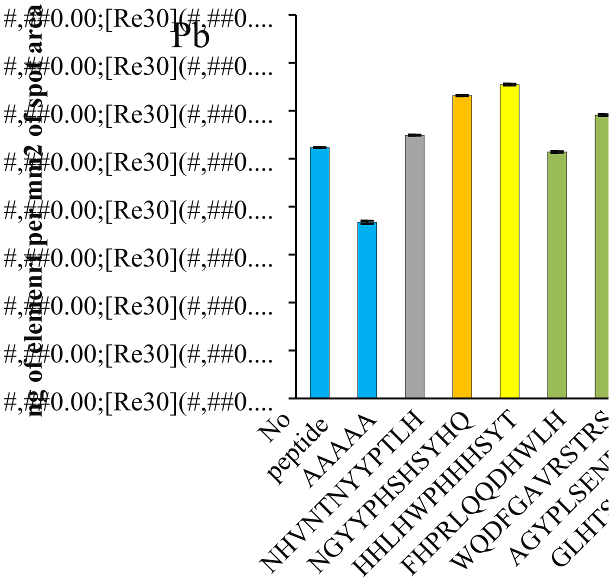

Figure S1. ICPMS analyses for Ti, Cr, Mn, Fe, Co, Ni, Cu, Zn, Y, La and Pb. (Continued)

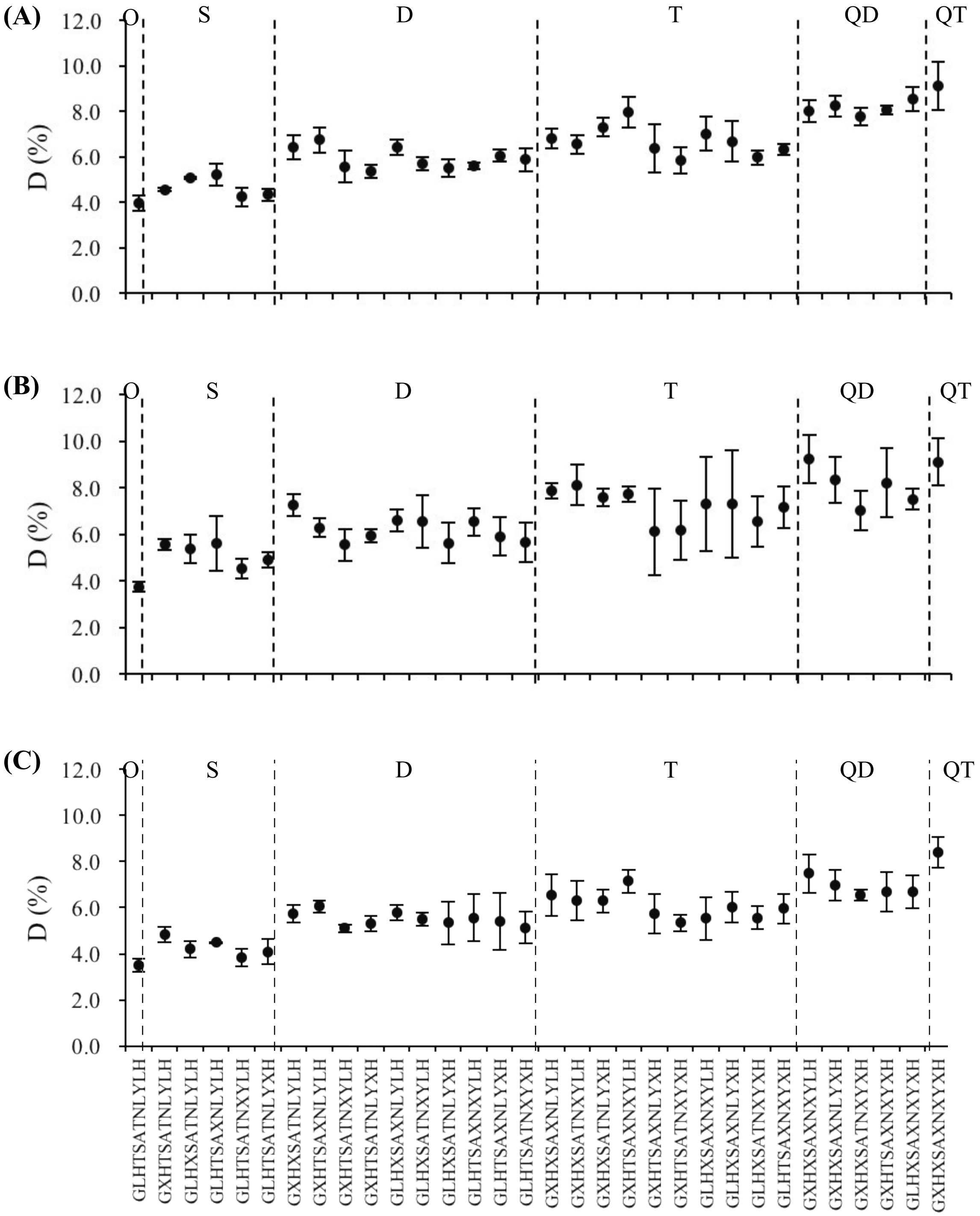

Figure S2. D values of substituted peptides, where X represents the substituted amino acid. (A) H substitution. (B) R substitution. (C) K substitution. O = Original, D = Double substitution, T = Triple substitution, QD = Quadruple substitution, QT = Quintuple substitution.

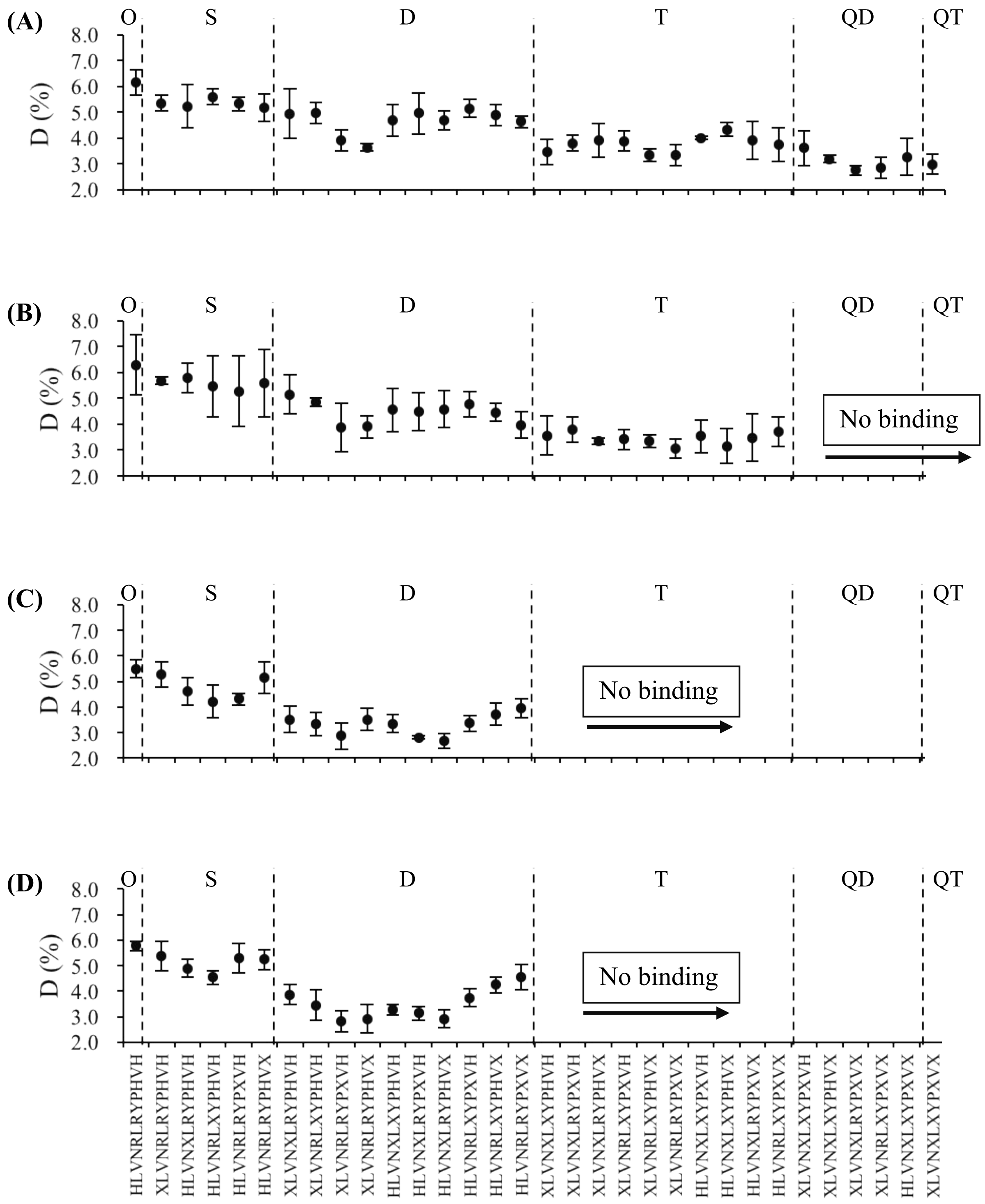

Figure S3. D values of substituted peptides, where X represents the substituted amino acid. (A) L substitution (B) T substitution (C) D substitution (D) E substitution. O = Original, D = Double substitution, T = Triple substitution, QD = Quadruple substitution.
